# Accurate pneumoconiosis staging via deep texture encoding and discriminative representation learning

**DOI:** 10.3389/fmed.2024.1440585

**Published:** 2024-10-09

**Authors:** Liang Xiong, Xin Liu, Xiaolin Qin, Weiling Li

**Affiliations:** ^1^Chengdu Institute of Computer Applications, Chinese Academy of Sciences, Chengdu, China; ^2^University of Chinese Academy of Sciences, Beijing, China; ^3^School of Computer Science and Technology, Dongguan University of Technology, Dongguan, China; ^4^Chongqing Prevention and Treatment Hospital for Occupation Diseases, Chongqing, China

**Keywords:** pneumoconiosis staging, chest X-ray, deep texture encoding, supervised contrastive learning, label distribution learning

## Abstract

Accurate pneumoconiosis staging is key to early intervention and treatment planning for pneumoconiosis patients. The staging process relies on assessing the profusion level of small opacities, which are dispersed throughout the entire lung field and manifest as fine textures. While conventional convolutional neural networks (CNNs) have achieved significant success in tasks such as image classification and object recognition, they are less effective for classifying fine-grained medical images due to the need for global, orderless feature representation. This limitation often results in inaccurate staging outcomes for pneumoconiosis. In this study, we propose a deep texture encoding scheme with a suppression strategy designed to capture the global, orderless characteristics of pneumoconiosis lesions while suppressing prominent regions such as the ribs and clavicles within the lung field. To further enhance staging accuracy, we incorporate an ordinal label distribution to capture the ordinal information among profusion levels of opacities. Additionally, we employ supervised contrastive learning to develop a more discriminative feature space for downstream classification tasks. Finally, in accordance with standard practices, we evaluate the profusion levels of opacities in each subregion of the lung, rather than relying on the entire chest X-ray image. Experimental results on the pneumoconiosis dataset demonstrate the superior performance of the proposed method confirming its effectiveness for accurate pneumoconiosis staging.

## 1 Introduction

**Figure 1:**
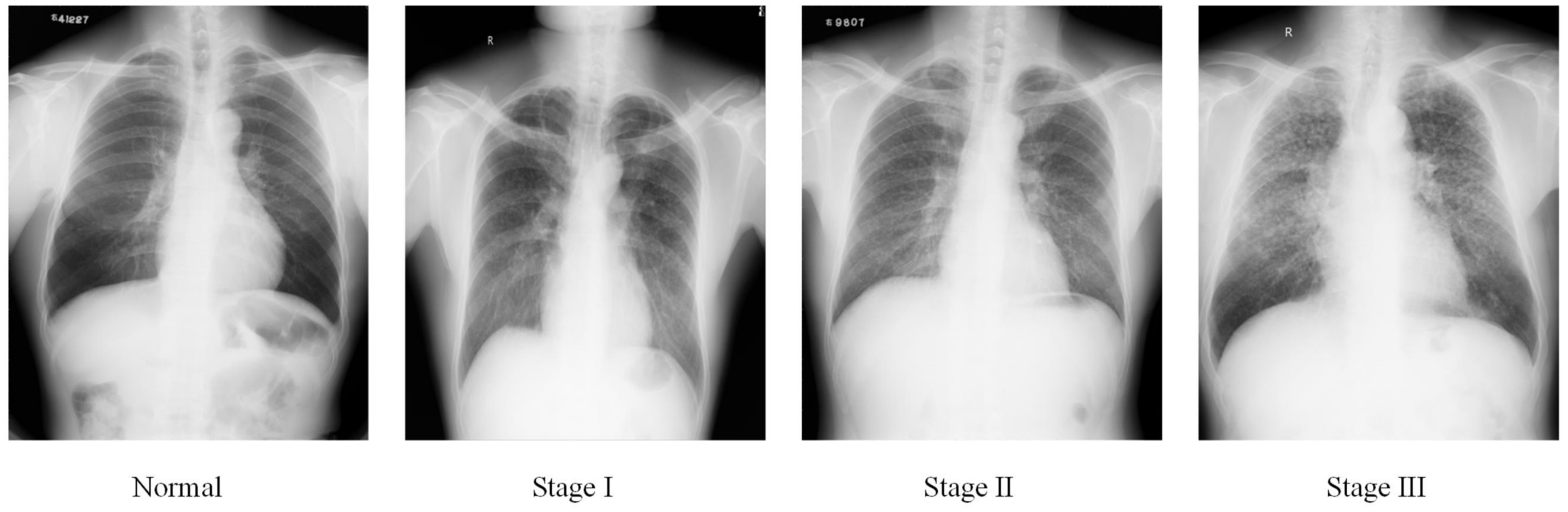
Examples of pneumoconiosis stage.

Pneumoconiosis is caused by the long-term inhalation of harmful dust particles, leading to lung fibrosis and inflammation (1). This condition permanently damages the patients’ respiratory system and weakens their physical strength. Accurate pneumoconiosis staging is crucial for facilitating early intervention and providing necessary welfare protection for affected individuals. At present, pneumoconiosis staging in clinical settings is conducted by well-trained radiologists who visually identify abnormalities on chest radiography, following guidelines established by organizations such as the International Labor Organization (ILO) (2) and the National Health Commission of China (NHC). Based on the shape, density, and distribution of pneumoconiotic opacities, the staging results are categorized into stages I, II, and III, as illustrated in Figure 1.

**Figure 2:**
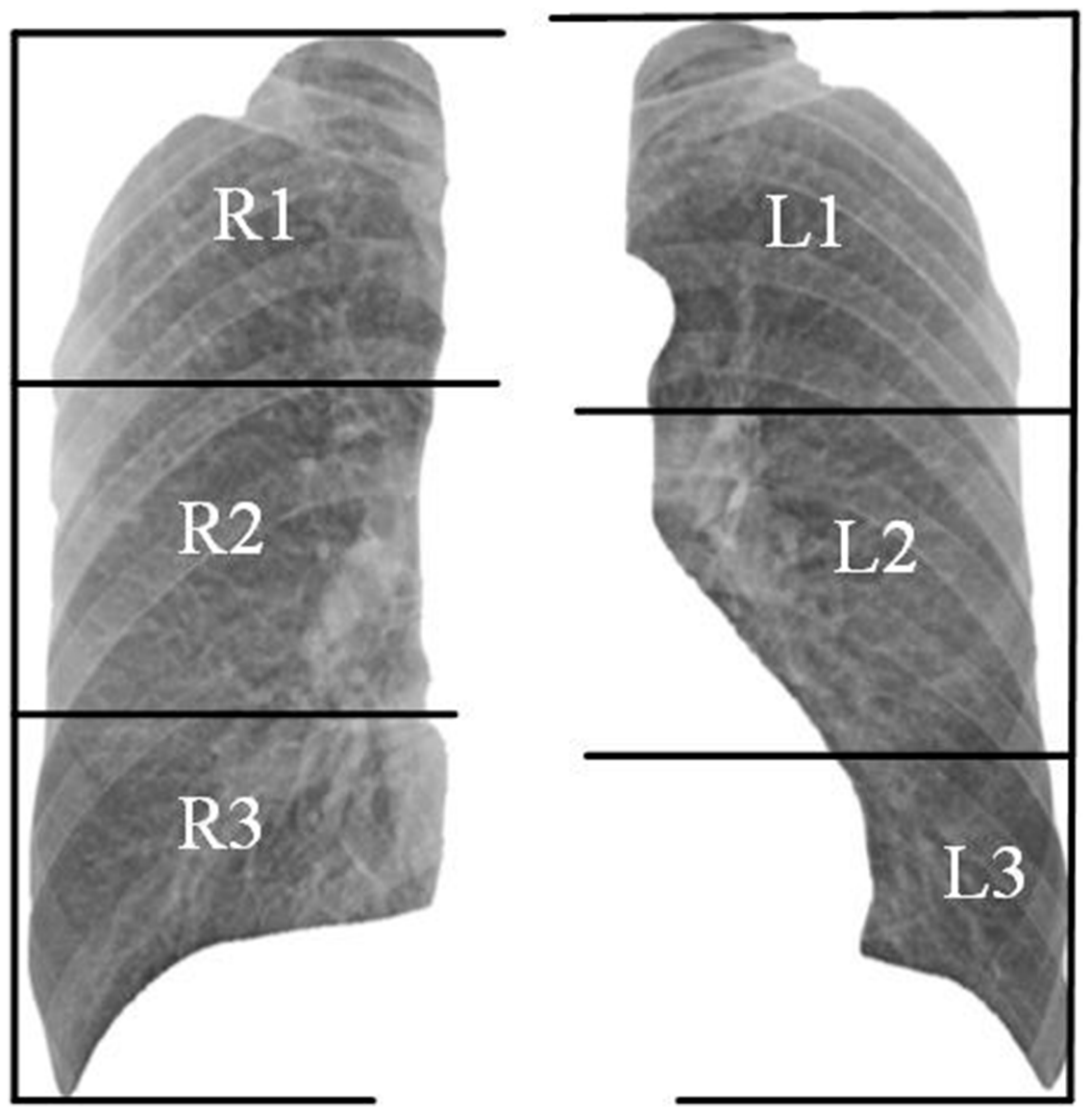
Example of subdivision of the lung fields.

Organizations such as the ILO and NHC have developed standardized pneumoconiosis staging standards to guide the diagnostic process. These protocols provide a systematic approach. First, the lung field is divided into six subregions, as shown in Figure 2. Second, authorized diagnostic radiologists evaluate the profusion level of opacities in each subregion and categorize them into levels 1, 2, or 3. Finally, the overall pneumoconiosis stage is determined by summarizing the subregion-based results according to the criteria outlined in Table 1. However, this manual process is labor-intensive and prone to significant inter- and intra-rater variability. As a result, there is an urgent need for a computer-aided diagnosis system that adheres to these standards to evaluate pneumoconiosis staging more efficiently and consistently.

**Table 1:** Final staging decision based on GBZ70-2015.

Stage	Description
Normal	No opacities or profusion level 1 of opacities occurred in one subregion.
Stage I	Profusion level 1 of opacities occurred in more than two subregions.
Stage II	Profusion level 2 of opacities occurred in more than four subregions, or profusion level 3 of opacities occurred in four subregions.
Stage III	Profusion level 3 of opacities occurred in more than four subregions, and opacity aggregation occurred.

Building on the remarkable success of deep learning in computer vision, recent methods (3, 4) have applied deep convolutional neural networks (CNNs) to improve the accuracy of pneumoconiosis staging. However, several challenges remain:

(1) Most previous studies treat pneumoconiosis staging as a simple image classification task, using chest radiographs and their staging results as input to train the classification model. However, this approach neglects the fundamental role of evaluating the profusion levels of opacities in subregions, which is essential for determining the final stage.
(2) Opacities of various sizes are not concentrated in a single region but are randomly dispersed throughout the lung field, showing a global orderless texture. The convolutional layer of CNNs functions as local feature extractors using a sliding window approach, which preserves the relative spatial arrangement of the input image in the output feature maps. While this is effective for recognizing large, whole objects, it is less suitable for classifying fine-grained medical images that require global, orderless feature representation.
(3) Additionally, since pneumoconiosis lesions are fine-grained, statistical-based representation learning models may be distracted by prominent but irrelevant regions, such as ribs and clavicles, leading to overfitting, especially in low-data scenarios.
(4) Pneumoconiosis also progresses gradually, with the number of opacities increasing over time. As a result, the profusion levels of opacities are not independent but follow an ordered sequence. However, traditional single-label learning overlooks the correlations between adjacent grades.
(5) To address these challenges, we propose a framework that adheres to standards for evaluating the subregion’s profusion levels of opacities in specific lung subregions on chest films. First, we construct a deep texture encoding scheme with a suppression strategy to learn the global, orderless characteristics of pneumoconiosis lesions while suppressing prominent regions such as the ribs and the clavicles in the lung field. Then, based on the observation that pneumoconiosis is a gradual process and similar texture images of pneumoconiosis tend to be grouped into close profusion levels, we adopt label distribution learning (5) to take advantage of the ordinal information among classes. Additionally, we employed supervised contrastive learning (6) to obtain a discriminative feature representation for downstream classification.

The main contributions of our study are as follows:

(1) We used a tailored deep texture encoding and suppressing module to extract the global orderless information of pneumoconiosis lesions in chest X-rays.
(2) We adopted label distribution learning to leverage the ordinal information among profusion levels of opacities and use supervised contrastive learning to obtain a discriminative feature representation for downstream classification.
(3) Extensive experiments on the pneumoconiosis dataset show the superior performance of the proposed method.

## 2 Related studies

The study of computer-aided diagnosis of pneumoconiosis dates back to the 1970s. It can be categorized into traditional machine learning methods and deep convolutional neural network methods. The traditional methods extract shallow features from chest X-rays and then pass the features to a classifier for classification. Savol et al. (7) investigated the AdaBoost model to distinguish small, rounded pneumoconiosis opacities based on image intensity.

Zhu et al. (8) trained a Support Vector Machine (SVM) classifier using a wavelet-based energy texture feature, showing good potential for detecting and differentiating pneumoconiosis stage I and II from normal. Yu et al. (9) adopted an SVM classifier to diagnose pneumoconiosis by extracting the texture feature of chest X-rays by calculating the statistical characteristics of the gray-level co-occurrence matrix. Okumura et al. (10) employed power spectra and artificial neural networks for pneumoconiosis staging. In a later study, Okumura et al. (11) proposed a three-stage network to improve classification accuracy. These traditional methods with handcrafted feature extractors benefit from model interpretability. However, the diagnostic accuracy of these methods has not yet met practical requirements. Recently, CNN-based methods have successfully diagnosed pneumoconiosis and staging by learning complex features automatically. Wang et al. (12) analyzed the character of pneumoconiosis and identified it with Inception-V3, achieving an AUC of 0.878. Devnath et al. (3) adopted an ensemble learning method with features from the CheXNet-121 model to detect pneumoconiosis included in 71 CXRs and obtained promising results. Zhang (13) explored a discriminant method for pneumoconiosis staging by detecting multi-scale features of pulmonary fibrosis trained with the Chest X-ray14 data set and a self-collected data set including 250 pneumoconiosis DR samples. To solve the problem that training data is insufficient, Wang et al. (14) augmented the data set using a cycle-consistent adversarial network to generate plentiful radiograph samples. Then, they trained a cascaded framework for detecting pneumoconiosis with both real and synthetic radiographs. Yang et al. (4) proposed a classification model with ResNet34 as the backbone to stage 1,248 pneumoconiosis digital X-ray images. The accuracy of diagnosis is 92.46%, while 70.1% is in grading of pneumoconiosis. According to the routine diagnostic procedure, Zhang et al. (15) developed a two-stage network for pneumoconiosis detection and staging. The accuracy for staging surpasses 6% higher than that gained by two groups of radiologists. To alleviate the problem of model overfitting caused by noisy labels and stage ambiguity of pneumoconiosis, Sun et al. (16) proposed a full deep learning pneumoconiosis staging paradigm that comprised an asymmetric encoder-decoder network for lung segmentation and a deep log-normal label distribution learning method for staging, and achieved accuracy and AUC of 90.4 and 96%, respectively.

## 3 Methods

**Figure 3:**
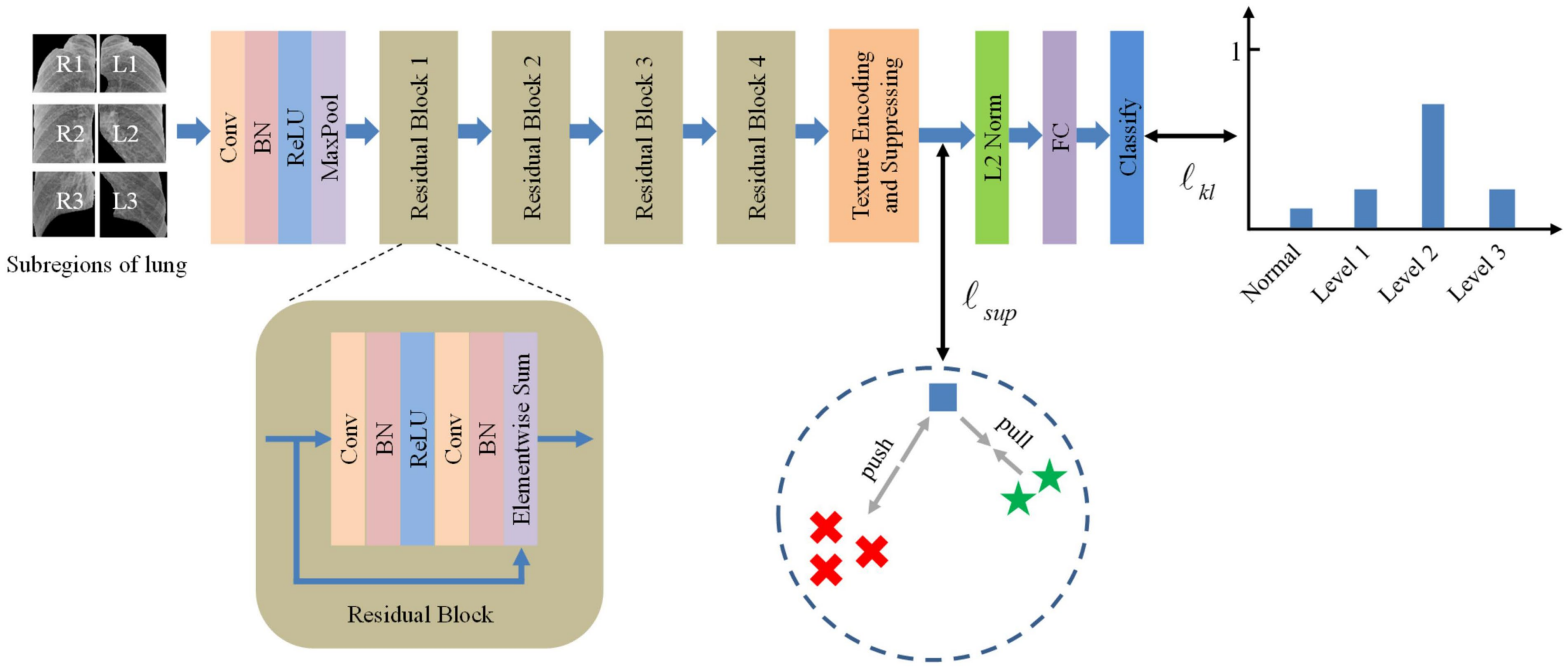
The overall framework of the proposed method.

Inspired by the deep texture encoding network (17), which has proven to be efficient in texture/material recognition, we construct a tailored model for pneumoconiosis staging via deep texture extracting and discriminative representation learning. The overall architecture of the model is shown in Figure 3. A ResNet (18) network is employed as a feature extractor.

Then, features are fed into the texture encoding and suppressing module (TES) to obtain a global orderless representation of texture details.

The outputs from the TES module are supervised using contrastive learning, followed by a fully connected layer for classification via label distribution learning.

### 3.1 Texture encoding and suppressing

Texture encoding integrates the whole dictionary learning and visual encoding to provide a global, orderless representation of texture information. Here, we briefly introduce prior work for completeness. Given a feature map $X\in {\mathbb{R}}^{W\times H\times C}$ , where *W*, *H*, and *C* are the width, height, and channel, it can be expressed as $X=\left\{x_{1},\ldots ,x_{N}\right\}$ , where *N=W × H*. The codebook $D=\left\{d_{1},\ldots ,d_{K}\right\}$ contains *K* learnable codewords. The corresponding residual vector of the feature map X is computed by $r_{ij}=x_{i}-d_{j}$ , where $x_{i}\in {\mathbb{R}}^{1\times C}$ , i=1,…,N and $d_{j}\in {\mathbb{R}}^{1\times C}$ , j=1,…,K . The residual encoding for codeword $d_{j}$ can be expressed by the following Equation 1:


(1) $$e_{j}=\overset{N}{\underset{i=1}{\sum }}e_{ij}=\overset{N}{\underset{i=1}{\sum }}\omega_{ij}r_{ij}\text{,}$$

where the weight $\omega_{ij}$ for the residual vector $r_{ij}$ is obtained by Equation 2:


(2) $$\omega_{ij}=\frac{e^{-s_{j}{r_{ij}}^{2}}}{\sum_{k=1}^{K}e^{-s_{k}{r_{ik}}^{2}}}\text{,}$$

where $S=\left\{s_{1},\ldots ,s_{K}\right\}$ are the learned smooth factors.

Therefore, a set of encoding vectors $E=\left\{e_{1},\ldots ,e_{K}\right\}$ is generated to represent the global orderless features of the feature map X .

Due to the similar anatomical structure of chest X-rays and the fine-grained nature of pneumoconiosis lesions, we employ a suppression strategy (19) to diminish the prominence of salient regions such as ribs and clavicles, encouraging the network to focus on opacities that are critical for pneumoconiosis staging. Let $P_{l}\in {\mathbb{R}}^{K}$ be the non-maximal vector corresponding to the *l*th channel of residual encoding $E_{l}$ . The form can be expressed by the following Equation 3:


(3) $$P_{l}\left(j\right)=\{\begin{array}{cc} \lambda , & \mathrm{if}E_{l}\left(j\right)=\max\left(E_{l}\right)\\ 1, & \mathrm{otherwise} \end{array}$$

where l=1,⋯C , $\max\left(E_{l}\right)$ is the maximum value of $E_{l}$ and λ is the suppression factor.

Then, we suppress the maximal value in the *l*th channel of residual encoding by the following Equation 4:


(4) $${\tilde{E}}_{l}=P_{l}\;ο\;E_{l}\text{,}$$

where ο represents element-wise multiplication.

### 3.2 Supervised contrastive learning

Supervised contrastive learning (6) allows us to effectively exploit label information by extending traditional contrastive loss, thereby learning a discriminative feature representation by pulling together samples of the same class and pushing apart those of different classes in embedding space. Specifically, let us denote a batch of samples with *C* classes as $Μ=\overset{C}{\underset{c=1}{\cup }}M_{c}$ , here, $M_{c}$ is the subset belonging to a single class and $\left|M_{c}\right|$ is its cardinality. The supervised contrastive loss can be calculated by the following Equation 5:


(5) $${𝓁}_{\text{sup}}=-\overset{C}{\underset{c=1}{\sum }}\frac{1}{\left|M_{c}\right|}\underset{i\in M_{c}}{\sum }\log\frac{\sum_{m\in M_{c}\backslash i}e^{sim\left(z_{i},z_{m}\right)/\tau }}{\sum_{j\in Μ\backslash i}e^{sim\left(z_{i},z_{j}\right)/\tau }}$$

where $z_{i}$ denotes the residual encoding vectors of the *i*th sample in subset $M_{c}$ , $z_{m}$ represents the residual encoding vector of a sample in subset $M_{c}$ where the *i*th sample is exclusive, $z_{j}$ denotes the residual encoding vector of a sample in the batch M where the *i*th sample is exclusive, $sim\left(\cdot \right)$ is the cosine similarity function, and τ is a scalar temperature parameter.

### 3.3 Label distribution learning

Traditional classification models usually use single-label learning to guide model training. Label distribution learning (5) is proposed to solve the issue of label ambiguity, e.g., age estimation (20), emotion classification (21), and acne grading (22), which covers a certain number of labels and each label represents a different degree to describe the instance. Thus, this paradigm can leverage both the ground-truth label and its adjacent labels to provide more guidance for model learning. As the profusion levels of opacities of pneumoconiosis obey an ordered sequence, we utilize an ordinal regression method (23) to obtain the label distribution of an instance. In particular, let $\left\{r_{1},r_{2},\cdots ,r_{C}\right\}$ be the scope of profusion levels with ordinal sequence. Given a pneumoconiosis image $a_{i}$ labeled with profusion level $r_{t}$ , then the single ground-truth label can be transformed to a discrete probability distribution throughout the label range as Equation 6:


(6) $$y_{a_{i}}^{j}=\frac{e^{-|r_{t}-r_{j}|}}{\sum_{c=1}^{C}e^{-|r_{t}-r_{c}|}},\;j=1,2,\cdots ,C$$

where $y_{a_{i}}^{j}$ means the degree to describe the pneumoconiosis image $a_{i}$ by the *j*th profusion level.

For instance $a_{i}$ , the predicted probability distribution of each level can be expressed as Equation 7:


(7) $$p_{a_{i}}^{j}=\frac{e^{\theta_{j}}}{\sum_{m=1}^{C}e^{\theta_{m}}}$$

where $\theta_{j}$ denotes the predicted score concerning the *j*th profusion level outputted from the last FC layer.

We employ the Kullback–Leibler (KL) divergence to measure the information loss between the predicted and the ground-truth label distribution. Meanwhile, since there exists a serious class imbalance distribution, we use class reweighting (24) to deal with the imbalance problem of pneumoconiosis datasets, which is defined as the following Equation 8:


(8) $${𝓁}_{\mathrm{kl}}=-\frac{1}{\pi_{r_{t}}}\overset{C}{\underset{j=1}{\sum }}\left(y_{a_{i}}^{j}\mathrm{In}\frac{y_{a_{i}}^{j}}{p_{a_{i}}^{j}}\right)$$

where $\pi_{r_{t}}$ represents the label frequency of class $r_{t}$ in the training set.

### 3.4 Loss function

Therefore, the loss function for model training is the combination of supervised contrastive loss and label distribution loss. It can be formulated as the following Equation 9:


(9) $$L=\alpha {𝓁}_{\text{sup}}+\beta {𝓁}_{\mathrm{kl}}$$

where α and β are the hyperparameters.

At the testing stage, the label distribution of the given pneumoconiosis image can be predicted, where the label corresponding to the highest probability is considered the predicted profusion level of opacities.

## 4 Experiment

### 4.1 Datasets and evaluation metrics

It is important to note that a pneumoconiosis dataset that does not adhere to established standards, such as GBZ70-2015, has no practical value. Currently, there is no publicly available pneumoconiosis dataset that complies with GBZ70-2015. Therefore, we have collaborated with an occupational disease prevention and control hospital to construct a dataset that meets the GBZ70-2015 standard, with approval from the ethics committee. The dataset comprises 160 pneumoconiosis digital radiograph samples, each with a resolution of 2,304 × 2,880, and the profusion level of each subregion labeled by experienced radiologists. To increase the number of normal samples, we incorporated 93 chest X-rays of healthy patients from the public JSRT dataset (25), each with a resolution of 2048 × 2048.

According to the diagnostic standard, we first segmented the lung field using a pre-trained U-Net model (26), then divided it into six subregions, as shown in Figure 2. We removed the redundant background and resized each subregion to 256 × 256 pixels. Given the general symmetry of chest anatomy, we recombined the subregions of the left and right lungs to form data cases C1-C3. The statistics on the profusion levels of opacities for each data case are listed in Table 2.

**Table 2:** Statistics on profusion level of opacities in each data case.

Data cases	Level 0	Level 1	Level 2	Level 3
C1(L1 + R1)	225	157	90	34
C2(L2 + R2)	243	162	84	17
C3(L3 + R3)	327	122	52	5

In this study, accuracy (Acc), precision (Pre), sensitivity (Sen), and F1 score are used as evaluation metrics to assess the performance of each method. Accuracy refers to the proportion of correctly classified samples, precision indicates the proportion of correct predictions within the positive category, sensitivity (or recall) represents the model’s ability to detect positive category samples, and the F1 score balances precision and sensitivity to provide an overall measure of the model’s performance.

### 4.2 Implementation details

An instantiation of the proposed model is shown in Table 3. The size of input images is 256 × 256. The number of codewords is set to be 8. An 18-layer ResNet (18) with the global average pooling layer removed is used as a feature extractor, and a 1 × 1 convolutional layer is employed to reduce the number of channels. Then, output from the texture encoding and a suppressing module is supervised with contrastive learning, followed by L2 normalization and an FC layer for classification.

**Table 3:** Architecture of the proposed model based on ResNet18.

Layer Name	Filter Type	Output Size
conv1	7 × 7, stride 2	128 × 128 × 64
Res1	3 × 3 max pool, stride 2	64 × 64 × 64
	$\left[\begin{array}{c} 3\times 3,64\\ 3\times 3,64 \end{array}\right]\times 2$	
Res2	$\left[\begin{array}{c} 3\times 3,128\\ 3\times 3,128 \end{array}\right]\times 2$	32 × 32 × 128
Res3	$\left[\begin{array}{c} 3\times 3,256\\ 3\times 3,256 \end{array}\right]\times 2$	16 × 16 × 256
Res4	$\left[\begin{array}{c} 3\times 3,512\\ 3\times 3,512 \end{array}\right]\times 2$ $\left[1\times 1,256\right]\times 1$	8 × 8 × 256
Texture encoding	Eight codewords	8 × 256
Classification	FC	Four classes

The input image size is 256×256.

We implement all the methods using the Keras 2.6 framework and train the models with a GPU RTX 2080i. Adam optimizer with a learning rate of 0.0001 is used to optimize the networks. Batch size and epoch are set to be 16 and 300. Hyperparameters *α*, *β*, and *τ* are set to be 0.5, 0.5 and 0.1, respectively. Data augmentation approaches, including horizontal flipping and rotating (10 degrees), are used during the model training. Five-fold cross-validation is used for each experiment.

### 4.3 Ablation experiments

To thoroughly analyze the proposed model, we conducted ablation experiments on data case C1 to highlight the effectiveness of its critical components. Table 4 shows the results of the model with different components. From Table 4, we observe that compared to the baseline model trained with the cross-entropy loss function, the model incorporating ordinal label distribution learning improves accuracy from 78.2 to 79.4% and the F1 score from 66.8 to 67.2%. These results indicate that multiple labels provide more informative guidance for model learning than single labels.

**Table 4:** Ablation studies for the proposed model on data case C1.

ResNet18(base)	TES	LDL	SCL	Acc	Pre	Sen	F1
√				0.782	0.687	0.640	0.668
√		√		0.794	0.690	0.654	0.672
√	√			0.826	0.705	0.657	0.701
√	√	√		0.833	0.726	0.693	0.709
√	√	√	√	0.849	0.742	0.718	0.730

TES represents deep texture encoding and suppressing modules. LDL denotes ordinal label distribution learning. SCL is supervised contrastive learning.

Compared to the baseline, the results presented in row 3 of Table 4 show that the addition of deep texture encoding and suppressing modules boosts accuracy by 4.4% and the F1 score by 3.3%. This significant improvement demonstrates that the TES module effectively extracts the texture features of pneumoconiosis lesions. The comparison between rows 3 and 4 of Table 4 indicates that incorporating label distribution learning further increases accuracy by 0.7% and the F1 score by 0.8%. When all modules are combined, the model’s accuracy and F1 score improve to 84.9 and 73.0%, respectively, proving the effectiveness of the proposed method for evaluating the profusion levels of opacities in lung subregions.

Similar to classic dictionary learning, increasing the number of learnable codewords *K* allows the encoding module to capture more texture details. The results for different codewords *K* are displayed in Table 5. It indicates that the model achieves the best performance when *K* = 8. The possible reason is that there are mainly different sizes of rounded opacities dispersing in the chest X-ray of pneumoconiosis except for the ribs and clavicles. The texture encoding module, with an appropriate number of codewords, can effectively aggregate these features.

**Table 5:** Accuracy with different codewords.

*K*	4	8	16	32
Acc	0.825	0.849	0.844	0.838

**Figure 4:**
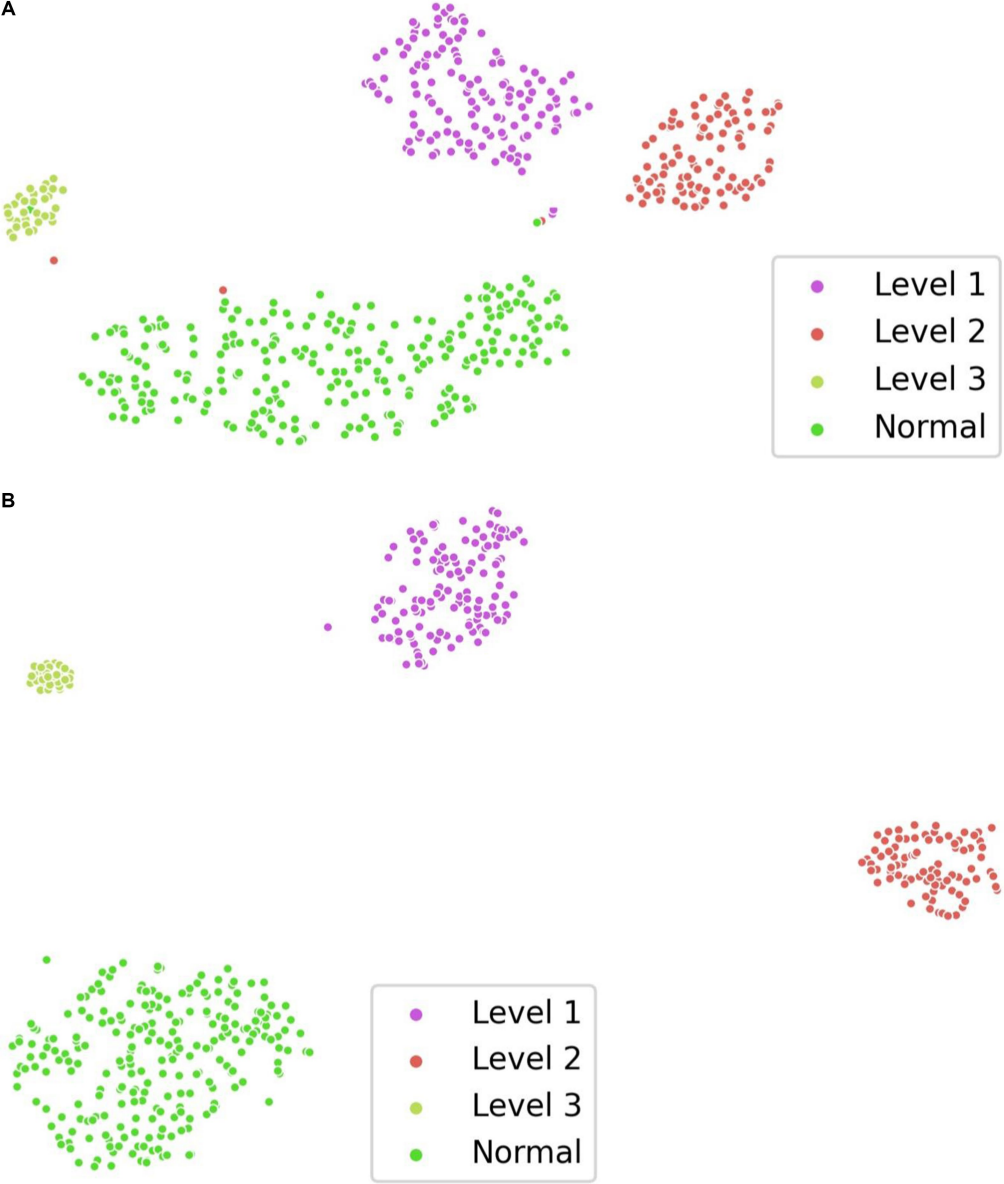
Visualization of feature representation distribution on data case C1. (A) w/o supervised contrastive learning. (B) w/ supervised contrastive learning.

To verify the effectiveness of supervised contrastive learning, we randomly selected 80% of the examples from data case C1 as the training set and visualized the distribution of feature representation output by the TES module using t-SNE (27). As shown in Figure 4, the model with supervised contrastive learning achieves a more discriminative representation, characterized by large interclass variations and small intraclass differences. Notably, the visualization shows a clear separation between normal images and images with profusion level 1, where lesions are subtle and difficult to detect.

**Figure 5:**
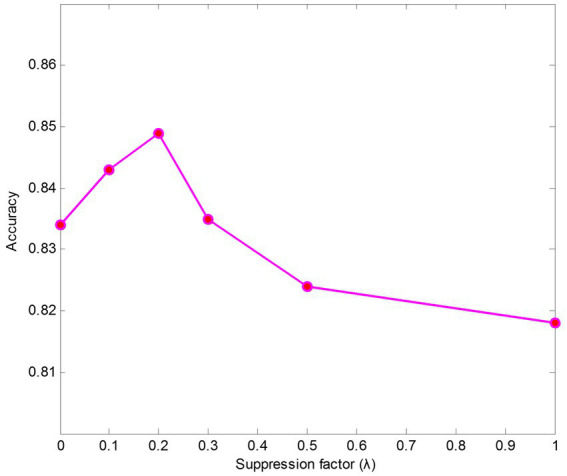
Accuracy with different suppression factors on data case C1.

We also conducted experiments on parameter sensitivity analysis of suppression factors λ . The results of different suppression factors are shown in Figure 5. The findings indicate that the model performs better when keeping λ a small value than it does without using a suppressing strategy ( λ = 1). The results also show that the texture encoding module can effectively aggregate salient regions’ features, such as the ribs and the clavicles. Specifically, λ=0.2 leads to the best performance on data case C1.

### 4.4 Comparison with the state-of-the-art methods

We compared the performance of the proposed method with five state-of-the-art image classification models based on CNN architectures. As shown in Table 6, the proposed method outperforms others in three of the data cases. It is important to note that while all the comparative models perform well in natural image classification tasks, they struggle with classifying texture-based images, such as those in the pneumoconiosis dataset.

**Table 6:** Comparison of different methods for data cases C1-C3.

Methods		Chollet (29)	Huang et al. (28)	Wang et al. (14)	Yang et al. (4)	Zhang et al. (15)	Ours
Backbone		Xception	DenseNet101	InceptionV3	ResNet34	ResNet101	Reset18
C1	Acc	0.753	0.781	0.749	0.785	0.778	0.849
	Pre	0.670	0.675	0.663	0.688	0.692	0.742
	Sen	0.613	0.624	0.582	0.634	0.619	0.718
	F1	0.646	0.651	0.625	0.658	0.653	0.730
C2	Acc	0.701	0.708	0.685	0.726	0.711	0.819
	Pre	0.660	0.679	0.676	0.680	0.676	0.702
	Sen	0.548	0.546	0.539	0.553	0.550	0.591
	F1	0.601	0.605	0.598	0.608	0.605	0.642
C3	Acc	0.690	0.693	0.682	0.696	0.685	0.776
	Pre	0.623	0.620	0.612	0.627	0.633	0.674
	Sen	0.527	0.535	0.519	0.538	0.526	0.575
	F1	0.570	0.573	0.565	0.579	0.577	0.621

We compared the performance of the proposed method with five state-of-the-art image classification models based on CNN architectures. As shown in Table 6, the proposed method outperforms others in three of the data cases. It is important to note that while all the comparative models perform well in natural image classification tasks, they struggle with classifying texture-based images, such as those in the pneumoconiosis dataset.

From Table 6, ResNet-based models (e.g., ResNet34, ResNet101) and DenseNet-based models (e.g., DenseNet101) generally outperform other methods. This may be due to the fact that networks with residual or dense connections are better at mitigating gradient vanishing during training, allowing them to retain more fine-grained details of small opacities associated with pneumoconiosis. However, models like Xception, DenseNet101, InceptionV3, and ResNet101, despite being deeper networks, do not show significantly improved performance. This suggests that the pneumoconiosis dataset lacks sufficient volume for these larger models to effectively learn the necessary features for classification.

Additionally, performance on C3 is the lowest across all methods. This is likely because the subregions in C3 represent a smaller lung area, resulting in fewer features available for extraction. A similar effect is seen with C2, where some lung areas overlap with the heart and hilum, further complicating feature extraction.

**Figure 6:**
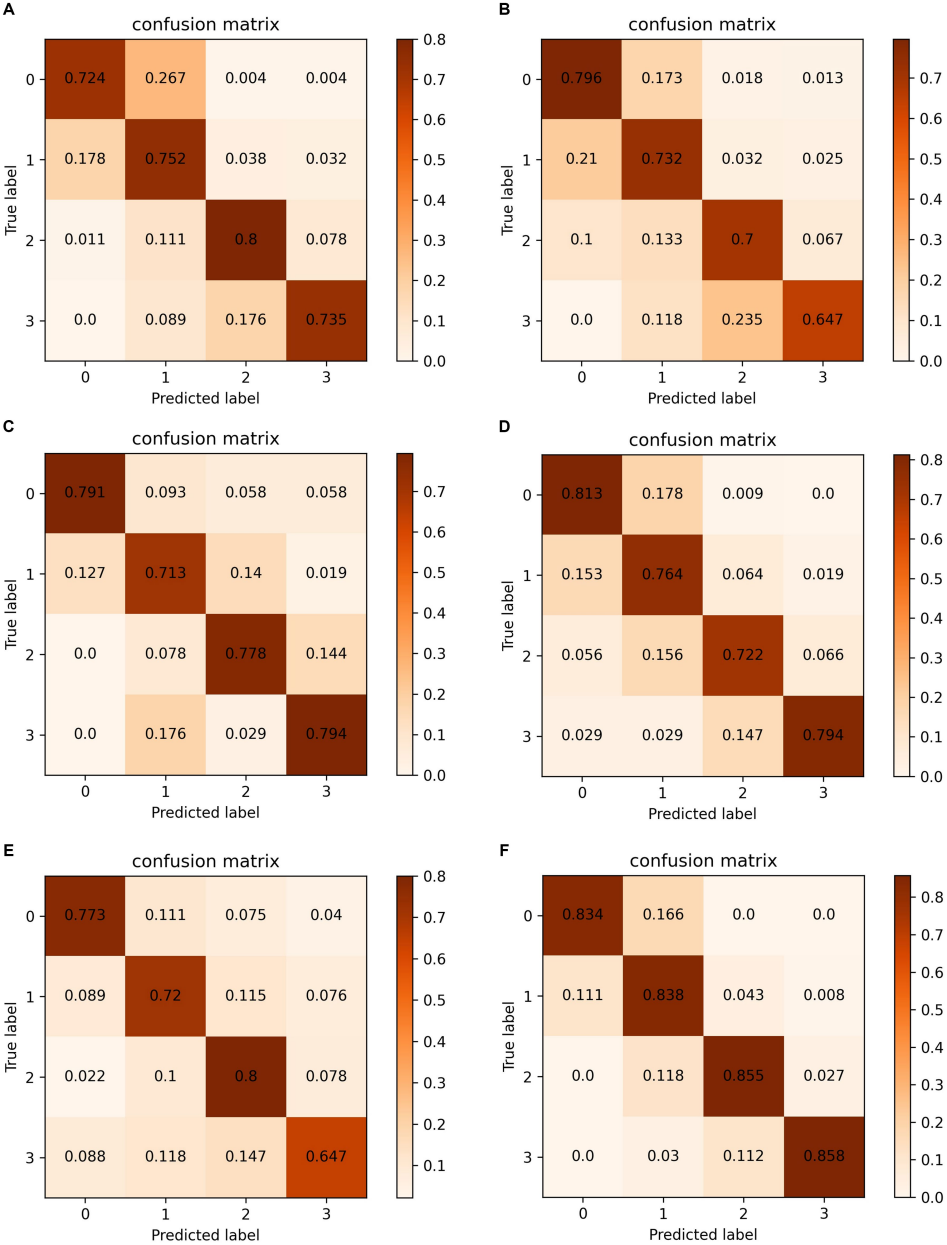
Confusion matrixes on data case C1. (A) Chollet (29). (B) Huang et al. (28). (C) Wang et al. (14). (D) Yang et al. (4). (E) Zhang et al. (15). (F) Ours.

To better illustrate the classification accuracy for each method, we provide confusion matrices for data case C1 in Figure 6. The diagonal elements of these matrices indicate the accuracy for each class. The proposed method achieves the highest accuracy and offers predictions that are closest to the ground truth labels, providing valuable insights for clinical diagnosis.

## 5 Conclusion

Accurate pneumoconiosis staging is a challenging task, as the staging result depends on the profusion level of opacities, which are randomly dispersed throughout the lung field. However, traditional CNNs struggle to directly learn the global, orderless feature representation of pneumoconiosis lesions. In this study, adhering to established standards, we propose a deep texture encoding scheme with a suppression strategy to evaluate the profusion levels of opacities in subregions. By incorporating label distribution learning to leverage the ordinal relationships among profusion levels, our approach achieves competitive performance. Additionally, the model enhances its feature representation through supervised contrastive learning. Experimental results on the pneumoconiosis dataset demonstrate the superior performance of the proposed method. A limitation of this study is the relatively small number of pneumoconiosis images. In future work, we aim to further improve staging accuracy by incorporating multi-modal imaging, such as X-rays and high-resolution CT scans.

## Data Availability

The data analyzed in this study is subject to the following licenses/restrictions: the datasets of this study are available from Chongqing Prevention and Treatment Hospital for Occupation Diseases but restrictions apply to the availability of these data, and so are not publicly available. However, it is available from the corresponding author on reasonable request. Requests to access these datasets should be directed to weilinglicq@outlook.com.

## References

[1] Wang T Sun W Wu H Cheng Y Li Y Meng F. Respiratory traits and coal workers’ pneumoconiosis: Mendelian randomisation and association analysis. Occup Environ Med. (2021) 78:137–41. doi: 10.1136/oemed-2020-106610, PMID: 33097673

[2] International Labor Organization (ILO). Guidelines for the use of the ILO international classification of radiographs of pneumoconiosis, Occupational Safety and Health Series, No. 22 (Rev.). Geneva Switzerland: International Labor Office (2011).

[3] Devnath L Fan ZW Luo SH Summons P Wang D. Detection and visualisation of pneumoconiosis using an Ensemble of Multi-Dimensional Deep Features Learned from chest X-rays. Int J Environ Res Public Health. (2022) 19:11193. doi: 10.3390/ijerph191811193, PMID: 36141457 PMC9517617

[4] Yang F Tang ZR Chen J Tang M Wang S Qi W. Pneumoconiosis computer aided diagnosis system based on X-rays and deep learning. BMC Med Imaging. (2021) 21:189. doi: 10.1186/s12880-021-00723-z 34879818 PMC8653800

[5] Geng X. Label distribution learning. TKDE. (2016) 28:1734–48. doi: 10.1109/TKDE.2016.2545658

[6] Khosla P Teterwak P Wang C. Supervised contrastive learning. Proceedings of the 34th International Conference on Neural Information Processing Systems. (2020) 18661–73.

[7] Savol A Li C Hoy R. Computer-aided recognition of small rounded pneumoconiosis opacities in chest X-rays. IEEE Trans Pattern Anal Mach Intell. (1980) PAMI-2:479–82. doi: 10.1109/TPAMI.1980.6592371

[8] Zhu B Luo W Li B Chen B Yang Q Xu Y. The development and evaluation of a computerized diagnosis scheme for pneumoconiosis on digital chest radiographs. Biomed Eng Online. (2014) 13:141. doi: 10.1186/1475-925X-13-141, PMID: 25277489 PMC4271323

[9] Yu P Xu H Zhu Y Yang C Sun X Zhao J. An automatic computer-aided detection scheme for pneumoconiosis on digital chest radiographs. J Digit Imaging. (2011) 24:382–93. doi: 10.1007/s10278-010-9276-7 20174852 PMC3092047

[10] Okumura E Kawashita I Ishida T. Computerized analysis of pneumoconiosis in digital chest radiography: effect of artificial neural network trained with power spectra. J Digit Imaging. (2011) 24:1126–32. doi: 10.1007/s10278-010-9357-7, PMID: 21153856 PMC3222544

[11] Okumura E Kawashita I Ishida T. Computerized classification of pneumoconiosis on digital chest radiography artificial neural network with three stages. J Digit Imaging. (2017) 30:413–26. doi: 10.1007/s10278-017-9942-0 28108817 PMC5537088

[12] Wang X Yu J Zhu Q Li S Zhao Z Yang B. Potential of deep learning in assessing pneumoconiosis depicted on digital chest radiography. Occup Environ Med. (2020) 77:597–602. doi: 10.1136/oemed-2019-106386, PMID: 32471837

[13] Zhang Y. Computer-aided diagnosis for pneumoconiosis staging based on multi-scale feature mapping. Int J Comput Intell Syst. (2021) 14:191. doi: 10.1007/s44196-021-00046-5

[14] Wang D Arzhaeva Y Devnath L Qiao M Amirgholipour S Liao Q. Automated pneumoconiosis detection on chest X-rays using cascaded learning with real and synthetic radiographs In: 2020 digital image computing techniques and applications (DICTA) IEEE (2020).

[15] Zhang L Rong R Li Q Yang DM Yao B Luo D. A deep learningbased model for screening and staging pneumoconiosis. Sci Rep. (2021) 11:1–7. doi: 10.1038/S41598-020-77924-Z, PMID: 33500426 PMC7838184

[16] Sun W Wu D Luo Y Liu L Zhang H Wu S. A fully deep learning paradigm for pneumoconiosis staging on chest radiographs. IEEE J Biomed Health Inform. (2022) 26:5154–64. doi: 10.1109/jbhi.2022.3190923, PMID: 35834466

[17] Zhang H. Xue J. Dana K. (2017). “Deep TEN: texture encoding network.” In *Proceedings of the 2017 IEEE conference on computer vision and pattern recognition (CVPR)*, 2896–2905.

[18] He K. Zhang X. Ren S. Sun J. (2016). “Deep residual learning for image recognition.” *Proceedings of the IEEE conference on computer vision and pattern recognition*, 770–778.

[19] Xiao G Wang H Shen J Chen Z Zhang Z Ge X. Synergy factorized bilinear network with a dual suppression strategy for brain tumor classification in MRI. Micromachines. (2022) 13:15. doi: 10.3390/mi13010015, PMID: 35056179 PMC8780069

[20] Gao B. Zhou H. Wu J. Geng X. (2018). Age estimation using expectation of label distribution learning. In *The 27th international joint conference on artificial intelligence (IJCAI 2018)*. 712–718.

[21] Yang J. Sun M. Sun X. (2017). “Learning visual sentiment distributions via augmented conditional probability neural network.” *Proceedings of the Thirty-First AAAI Conference on Artificial Intelligence*. 224–230.

[22] Wu X. Wen N. Liang J., (2019). “Joint acne image grading and counting via label distribution learning.” In *2019 IEEE/CVF international conference on computer vision (ICCV)*. IEEE, 2019.

[23] Diaz R. Marathe A. (2019). “Soft Labels for Ordinal Regression.” In *2019 IEEE/CVF conference on computer vision and pattern recognition (CVPR)*. IEEE, 2019.

[24] Ren J Yu C Sheng S. Balanced meta-softmax for long-tailed visual recog-nition. Adv Neural Inf Proces Syst. (2020). doi: 10.48550/arXiv.2007.10740

[25] Shiraishi J Katsuragawa S Ikezoe J Matsumoto T Kobayashi T Komatsu KI. Development of a digital image database for chest radiographs with and without a lung nodule: receiver operating characteristic analysis of radiologists' detection of pulmonary nodules. Am J Roentgenol. (2000) 174:71–4. doi: 10.2214/ajr.174.1.1740071 10628457

[26] Ronneberger O. Fischer P. Brox T. (2015). “U-net: convolutional networks for biomedical image segmentation.” In *International Conference on Medical image computing and computer-assisted intervention*, 234–241.

[27] Van der Maaten L Hinton G. Visualizing Data using t-SNE. J Mach Learn. (2008) 9:2579–605.

[28] Huang G. Liu Z. Van Der Maaten L. (2017). “Densely connected convolutional networks.” In *Proceedings of the IEEE conference on computer vision and pattern recognition*, 2261–2269.

[29] Chollet F. (2017). “Xception: deep learning with depthwise separable convolutions.” In *Proceedings of the IEEE conference on computer vision and pattern recognition*, 1800–1807.

